# In Vitro Supplementation of Copper Modulates the Functional Th1/Th2 Phenotype of Peripheral Blood Mononuclear Cells in Cattle

**DOI:** 10.3390/ani11092739

**Published:** 2021-09-19

**Authors:** Michaela Bunting, Bethan Challice, Amanda Gibson, Steven van Winden

**Affiliations:** 1 Pathobiology and Population Sciences, Royal Veterinary College, Hawkshead Lane, North Mymms, Hatfield AL9 7TA, UK; misha.bunting@gmail.com (M.B.); bethchallice@gmail.com (B.C.); amg39@aber.ac.uk (A.G.); 2 London School of Hygiene and Tropical Medicine, Keppel Street, London WC1E 7HT, UK; 3 Centre of Excellence for Bovine Tuberculosis, IBERS, Aberystwyth University, Penglais, Aberystwyth, Ceredigion SY23 3EE, UK

**Keywords:** cattle, paratuberculosis, MAP, Johne’s disease, copper, PBMC

## Abstract

**Simple Summary:**

This study investigated the association of copper levels and the appearance of blood monocytes, a white blood cell. One type of monocytes (M1) supports cellular immunity and the M2 monocyte helps the immune system through antibodies. Five samples of cow monocytes (PBMCs) were incubated in different levels of copper; 0, 4, 8 and 16 μM. After stimulation under three different conditions, we stained them for CD14 and CD16 to allow typing of the monocytes (M1 and M2). M1 function was also measured through nitric oxide (NO) production. The results showed a significant reduction in viability of the monocytes with increased copper (*p* < 0.001). Increasing copper resulted in more M1 type monocytes in cows older than 4 years (*p* = 0.001). CD14 expression affected both CD16 (M2) expression and NO production. For CD16 expression, there was a further significant negative effect of copper levels in cows older than 4 years, whereas NO was not affected by the varying copper levels. In our small sample, monocytes incubating in a higher copper environment showed a stronger M1 support for better cellular immunity containing intracellular infections more effectively. In the live animal low copper levels could possibly affect progression of a bacterial infection to clinical disease.

**Abstract:**

This study investigated the association of copper levels and monocyte plasticity between M1 (CD14^+^ CD16^−^) and M2 (CD14^−^ CD16^++^) phenotypes. Five samples of female bovine PBMCs were incubated in 0, 4, 8 and 16 μM copper and stimulated (PPD-A, TLR- 2 ligand (Pam_3_CSK_4_), or media alone) before they were washed and stained for cell surface expression analysis by flow cytometry. M1 function was measured through nitric oxide production using a Griess assay. Flow cytometry analysis showed a significant reduction in viability with increased copper (*p* < 0.001). Increasing copper had a significant impact on CD14 expression (*p* = 0.026) and in cows older than 4 years copper levels positively affected CD14 expression (*p* = 0.001), whereas in animals of four years or younger, Cu did not affect the CD14 expression (*p* = 0.701 and 0.939, respectively). CD14 expression affected both CD16 expression and NO production. For CD16 expression, there was a further significant negative effect of copper levels in cows older than 4 years, NO was not affected by varying copper levels. In our small sample, monocytes in the presence of a higher copper environment showed a stronger M1 support for better cellular immunity which might contain intracellular infections more effectively. To test this, a randomised clinical trial will be required to determine whether copper supplementation could prevent progression to Johne’s disease in MAP infected cows.

## 1. Introduction

Johne’s disease is a chronic inflammatory disease caused by *Mycobacterium avium subspecies paratuberculosis* (MAP) in the ileal mucosa of cattle [1]. Johne’s disease (JD) is the progressive clinical presentation phase of MAP infection. Understanding how to control JD is of relevance, as it incurs substantial economic losses in the dairy sector. In 2012 an estimate suggested the disease cost the US dairy industry over 200 million USD per year [2].

MAP is a facultative intracellular pathogen of macrophages, enabling the mycobacteria to evade the immune system for years at a time [1]. Cattle are typically infected by the oro-faecal route as neonates. Crossing the mucosal barrier is facilitated by Microfold (M) cells in Peyer’s patches, allowing the bacteria to translate across the epithelium and access macrophages [3]. MAP also affects the tight junctions of the mucosa to further increase permeability [4]. Within the macrophage, MAP survives by preventing acidification of the phagosome. In preventing maturation into a phagolysosome, which would be fatal to the bacteria, the bacteria are provided with a compartment within the macrophage which facilitates replication and survival [5]. In this way, MAP can survive undetected for years. During this time, the inflammatory response to the pathogen-associated antigens in the submucosa and mesenteric lymph nodes causes local inflammation and cell recruitment. Granulomas form over time and as disease progresses the lesions become large enough to block absorption of nutrients and the animal will present with clinical disease [6].

Macrophages differentiate from circulating monocytes, a subpopulation of peripheral blood mononuclear cells (PBMCs). Monocytes are the precursors of innate cell types such as myeloid dendritic cells and macrophages. Circulating monocytes migrate to tissues in response to infection or damage signals, then differentiate into tissue specific macrophages, such as intestinal macrophages [7]. MAP survives in macrophages and monocytes by preventing acidification of the phagosome and apoptosis [8]. A study on miRNA expression in MAP infected monocytes showed that the monocytes modified miRNA expression and actively prevented apoptosis via miR-150 [9]. In order to overcome this mycobacterial survival strategy, macrophages must induce a protective immune response to control and destroy the pathogen. This protective immune response is mediated by Type 1 Helper CD4^+^ Lymphocyte (Th1) biased T cell response, which initiates the production of IFNγ in response to MAP-specific antigens presented by activated monocytes [8]. The ability of the host immune system to control MAP infection is dependent on this Th1 biased response, typically characterised by IFN-γ and IL-2 cytokines [10]. Th1 bias directs the immune system to enhance macrophage activation by IFN-γ, inducing inflammation and cell-mediated immunity, whereas a Th2 bias typified by IL-4 supports humoral immunity and antibody production. MAP is an intracellular infection, therefore Th2 biased humoral responses are less effective against the pathogen and would not lead to MAP control and clearance [11]. Nitric oxide (NO) produced by classically activated (or M1) macrophages is considered a hallmark of proinflammatory antimicrobial macrophage responses, which contributes to bacterial control and clearance. Subclinical cases of MAP have been shown to have increased levels of NO in their macrophages [4]. In contrast, M2 macrophages are characterised mostly by reduced NO secretion, anti-inflammatory responses and increased tissue remodelling ability [12]. As Johne’s disease progresses, there is an unexplained switch from Th1 to Th2 responses, resulting in uncontrolled MAP growth and presentation of clinical disease [4]. Consequently, macrophages are not supported by IFN-γ, so the bacteria are no longer targeted by the cellular immune system or controlled by activated macrophages. At this stage, the animal will have detectable antibodies for MAP and will be shedding bacteria in the faeces to infect the next generation of hosts [6].

The presence of M1 macrophages would be indicative of Th1 bias and could allow better control of MAP. Measuring changes in certain surface marker expression on monocytes provides an insight into M1/M2 bias. CD14 and CD16 surface expression markers can be used to group monocyte population subsets into classical M1 and non-classical M2 populations [13]. In bovines, CD14^+^ CD16^−^ is defined as the M1 classical subset, the M2 non-classical subset is CD14^−^ CD16^++^, and the intermediate subset is CD14^+^ CD16^+^ [14]. Nutrient and micronutrient status may affect the Th1/Th2 bias, for instance it has been shown that a Copper (Cu) deficiency reduces the ability of cattle to produce IFNγ and TNFα by mononuclear cells [15,16]. In an assessment of function, NO production was also increased [17]. Cu deficiency is detrimental to the immune system, including increased susceptibility to bacterial infection and impaired macrophage activity through a reduced respiratory burst activity [18,19]. More specifically, in a recent field trial there was a correlation found between cows positive for Johne’s disease having lower blood Cu levels [20]. The question is whether lower circulating Cu in JD cows is due to reduced Cu absorption, or whether low Cu facilitates JD progressing in MAP infected cattle. We therefore sought to investigate whether Cu levels could influence monocyte phenotype through the level of CD14 and CD16 expression and function via measuring the NO production in vitro. Our working hypothesis is that monocytes in a low Cu environment have a preferred expression of phenotype and function that is associated with the switch from M1 to M2 representing a Th2 bias.

## 2. Materials and Methods

### 2.1. PBMC Preparation and Stimulation Assay

Five samples PBMCs from adult Brown Swiss cattle were selected. The cows were multiparous, lactating and aged between 3 and 6 years old; one cow was younger than 4 years of age, two cows were aged four years and two cows were older than four years. They were clinically healthy at the time of PBMC isolation and repeatedly tested serologically negative for MAP. It should be noted that due to this small study size, age stratification cannot be assumed absolute and requires further study with larger sample sizes. The PBMCs were thawed at 37 °C, washed and counted before suspension in complete media (RPMI 1640 + GlutaMAX (Gibco), 10% FCS (Fisher Scientific) and 1X penicillin/streptomycin (Gibco). Cells were adjusted 5 × 10^6^ cells/mL in complete media and 100 μL were added per well in a 96 well microtitre plate according to Table 1 below.

CuSO_4_ (VWR) was prepared in complete media at 2X final concentrations required and 100 μL of each solution was added to the appropriate wells to yield the exact experimental concentrations (0, 4, 8 and 16 μM). Cells were incubated for 24 h at 37 °C prior to stimulation. At 24 h post seeding and pre-stimulation, PBMCs were suspended and 20 µL removed for viability assessment. Cells were washed to remove complete media and suspended in 50 μL of Live/Dead Violet dye (1:1000, Invitrogen), and incubated at 4 °C for 30 min in the dark. Viability ranged from 80–90% and we concluded that Cu treatment resulted in at least 80% viable cells after 24 h of co-culture. Cells were then stimulated with complete media, PPD-A (Purified protein derivative from *Mycobacterium avium* ssp *avium*) at 250 IU/mL (National Institute for Biological Standards and Controls (NIBSC)), and TLR2 ligand Pam_3_CSK_4_ lipoprotein (Invivogen) at 100 ng/mL; 24 h after stimulation, supernatants were harvested post centrifugation and stored at −20 °C, for subsequent NO assay. Cells were stained in situ as detailed below

### 2.2. Staining PBMC for CD14 and CD16 Surface Expression

**Figure 1:**
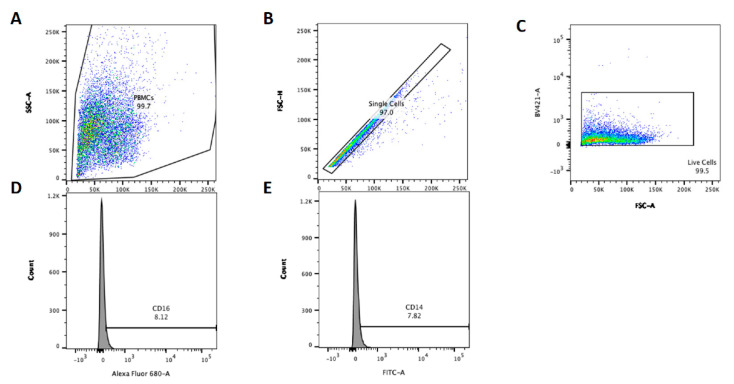
Example gating strategy for flow cytometry analysis in FlowJo software. Cells were adjusted for size and granularity, fluorescent parameter PMT voltages adjusted to account for autofluorescence as standard practice and gated to contain PBMC (**A**). PBMCs were assessed using FSC-A and FSC-H to determine single cell population within PBMC and gated (**B**). Live single cells were then gated based on exclusion of the live/dead stain (**C**). Percentage CD14 (FITC) and CD16 (Al680) expression was determined for live cell for all samples (**D**,**E**).

After the supernatant was harvested, the cells were prepared for cell surface marker staining and FACS analysis. Cells were washed to remove complete media and suspended in 50 μL of Live/Dead Violet dye (1:1000, Invitrogen), and incubated at 4 °C for 30 min in the dark. Cells were washed twice with PBS before being suspended in 50 μL of antibody staining solution: mouse anti-human CD14 (TÜK4): FITC (1:5 dilution, Bio-Rad), mouse anti-human CD16 (KD1): Alexa Flour 647 (1:5 dilution, Bio-Rad) and 1% BSA (Promega). Antibodies and clones used have been used extensively in bovine cell surface staining protocols; please refer to Sopp. et al., 1997 and Boysen et al., 2008 for cross-reactivity and functional staining profiles [21,22]. Cells were incubated at 4 °C for 30 min in the dark before washing twice with PBS, cells were suspended in 4% paraformaldehyde (PFA) (Fisher Scientific) and fixed for 15 min. Finally, cells were washed twice with PBS and suspended in PBS for analysis with a BD LSR FACS machine (Becton Dickenson). Unstained cells for each animal were used to define acquisition settings and to create gates. Data were acquired using BD FACSDiva software (Becton Dickenson) with 10,000 live, single cell events were recorded per sample using the gating strategy and sample set up outlined in Figure 1. The data were further analysed in FlowJo software (Becton Dickenson) using the gating strategy present in Figure 1 below to determine the percentage CD14 or CD16 positive PBMC. Population percentages were exported from FlowJo to Excel for comparisons between conditions. Relative CD14 and CD16 staining are denoted CDχχ^+^ and CDχχ^++^ to represent positive and bright positive staining, respectively.

### 2.3. Nitric Oxide (Griess) Release Assay

Harvested supernatants were evaluated indirectly for the presence of Nitric Oxide (NO) using the Griess Assay and a Nitrite standard curve. For each sample, 50 µL of cell culture supernatants were incubated with 50 µL of Griess reagent (containing equal volumes of solution A and B) in a microtitre plate. After 10 min the samples were analysed against a nitrate standard curve using a multimode plate reader (Tecan M200) set to measure absorbance at 550 nm. Sample data were then converted to µM nitrite using the standard curve (correlation coefficient of over 98%) in Excel.

### 2.4. Data and Statistical Analysis

The actual percentage of CD14 and CD16 expression and the percentage of cells alive in each condition was exported from FlowJo and processed in MS Excel. As NO production depends on the number of cells in the well, we normalised for viability.

The data were processed by a mixed linear model (MLM) in SPSS with cow as the random variable, to account for repeated measures. NO production, CD14 and CD16 expression were evaluated as dependent variables. Fixed effect parameters included age, Cu concentration in the media, stimulation treatment (PPD-A or TLR2 Ligand, or no stimulation), percentage survival and CD14 and CD16 expression.

A univariate approach was used to assess whether treatment or percentage survival influenced the dependent variables. Survival was significant and used in the multivariate approach. Treatment was not associated with the dependent variables and therefore not included in further analysis. As there may be a negative or positive feedback occurring with higher or lower CD14 or CD16 expression, they were used as explanatory variables in each other’s MLM. NO production had both CD14 and CD16 expression in the initial model. A multivariate approach was taken with all parameters in the model, removing the least significant parameter at each step, until all parameters were significant (*p* < 0.05). All parameters were put in as main effects and in addition Cu and Age were put in as an interaction.

The general formula for the model is expressed as:

y = β_0_+ β_1_*(surv) + β_2_*(CD14) + β_3_*(CD16) + β_4_*(Cu) + β_5_*(age) + (β_4_*(Cu)*β_5_*(age)) + u*(Z) + ϵ,

where β_0_ is the intercept, β_1–5_ are the coefficients of fixed effects; u is coefficient of random effect, and ϵ is the random error. All fixed effects were inputted as linear covariates.

y: CD14 expression (%), CD16 expression (%), and NO production (μM)

surv: Survival (%, range: 7.3–62.9)

CD14: CD14 expression (%, range: 1.9–21.0)

CD16: CD16 expression (%, range: 1.7–43.9)

Cu: Copper levels (μM, 0, 4, 8, and 16)

age: Age of the cows (years, range: 3.1–6.3)

Z: Random effect of 5 individual cows.

After completing the stepwise process, the final models were reported.

## 3. Results

After 24 h incubation and a further 24 h of stimulation as assessed by FACS, the average survival rate of PBMC was 39.2% (95% confidence interval: 35.6–42.8), CD14 expression 6.1% (95% CI: 5.0–7.2), CD16 expression 9.6% (95% CI: 6.6–12.5) and normalised NO production was 2.3 μM (95% CI: 1.7–2.9).

### 3.1. High Concentration Copper Treatment of PBMC Reduced Cell Viability

The type of cell stimulant (media alone, PPD-A or Pam_3_CSK_4_) during incubation did not affect CD14 or CD16 expression, nor the NO production. However, survival of PBMC was associated with these parameters (*p* < 0.01). PBMC stimulant at incubation did not affect the percentage of surviving cells (*p* = 0.079). Increasing Cu concentration in the media, however, significantly reduced viability at a rate of 0.9% per μM (*p* < 0.001). This justified including percentage of survival in the MLM models described below, so that the possible effect of Cu through survival rate was controlled for.

### 3.2. Effects of Copper on CD14 and CD16 Expression, and NO Production

Controlled for survival there was no univariate association between age and CD14, CD16 expression and NO production (*p* = 0.275, 0.278 and 0.051, respectively). Furthermore, there was no univariate association between Cu and CD14, CD16 expression and NO production (*p* = 0.804, 0.309 and 0.442, respectively). The backwards stepwise multivariate approach yielded significant association, and these are summarised in Table 2 below.

**Figure 2:**
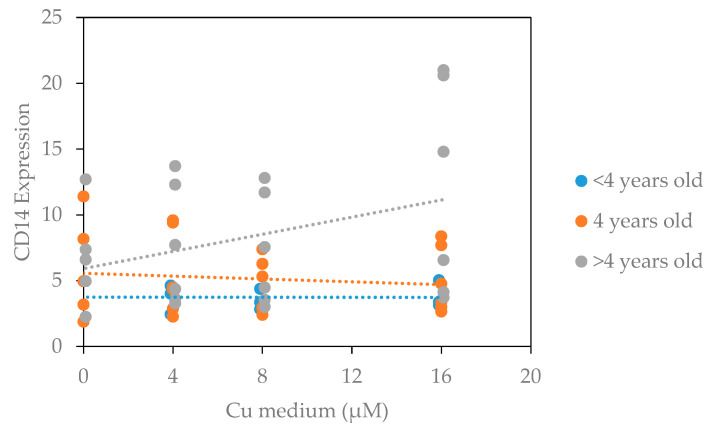
Association of copper levels and age on CD14 expression. PBMC incubated in the presence of increasing copper were exposed to different stimulations (media alone, PPD-A and Pam_3_CSK_4_) for 24 h, generating 3 data points for each cow. One cow was less than 4 years, two cows aged 4 years and two cows were older than 4 years of age. Expression of CD14 and CD16 on viable monocytes were determined by flow cytometry, analysed in FlowJo and then exported to Excel and further processed in a mixed linear model using SPSS.

In addition to a positive association with CD16 expression and survival rate, the CD14 expression was affected by Cu levels in the media. This association was stratified by age: in cows over four years of age Cu was positively associated with CD14 expression (Figure 2: 0.24 per μM of Cu, *p* = 0.001), whereas in animals of four years or younger, Cu did not affect the CD14 expression (*p* = 0.701 and 0.939, respectively). Increased CD14 expression positively correlated with both CD16 expression and NO production (*p* < 0.001).

**Figure 3:**
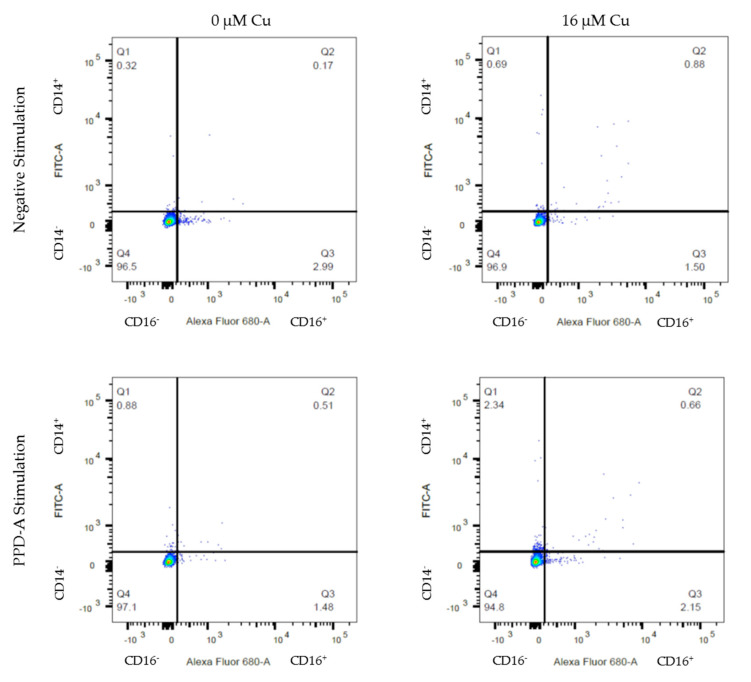
Representative flow cytometry graphs of PBMCs of a six year old cow in FlowJo software. Percentage CD14 (FITC) and CD16 (Al680) expression was determined for live cells in the presence of increasing copper (0 and 16 μM) and exposed to different stimulations (media alone and PPD-A) for 24 h.

In addition to the association with CD14 expression, Cu appeared to have a negative correlation with CD16 expression, as exemplified by Figure 3 below that shows the percentage of CD14 (FITC) and CD16 (Al680) expression was determined by FACS. This association was stratified by age: in animals of four years or younger, Cu did not affect the CD16 expression (*p* = 0.762 and 0.210, respectively), whereas in cows over four years of age, Cu was negatively correlated with CD16 expression: −0.30 per μM of Cu (*p* = 0.034). Nitric Oxide production was significantly associated with survival percentage and CD14 expression, but no further associations (Cu, age or CD16 expression) were significant.

## 4. Discussion

We explored whether monocytes in a low Cu environment would present an M2 phenotype (CD14^−^ CD16^++^), and monocytes in a high Cu environment would present an M1 phenotype (CD14^+^ CD16^−^) on the basis that M1 phenotype would be protective against MAP infections progressing in Johne’s disease. Our data suggest that increasing Cu from 0 to 16 μM in PBMCs from older (>4 years) dairy cow donors had a positive correlation with CD14 expression and this association was not apparent in younger cows. Equally, in older cows CD16 expression appeared to be negatively correlated with increased Cu levels, as exemplified by Figure 3, possibly indicative of a M2 to M1 switch. The CD16 expression positively correlated with CD14 levels, suggesting that the PBMC populations under study here were mainly operating as Intermediate Monocytes [13,14,23]. In our NO assay, we could not find an additional association of copper levels and NO production. The multivariate regression suggests variation in NO production was mainly through the changed expression of CD14. This shows that the function of the PBMCs follows the phenotypical presentation. Both CD16 expression and NO production were affected by CD14 expression, placing a central role of CD14 in the dynamics of our observations of the PBMCs.

The donor cows had no evidence of MAP infection, so we assume these are non-JD specific dynamics. It will be important to evaluate whether the Cu-Th1/Th2 relationship is different in cows with different stages of MAP infection: non-infected, containing the pathogen, or progressing into disease. Our results indicate that there could be an age specific Cu dependency of the Th1/Th2 PBMC phenotype, potentially occurring in older cows. This is an important area for further study as, coincidently, MAP infected cows around 4 years old tend to start progressing into Johne’s disease [6]. Our current in vitro results cannot confirm a role of Cu on controlling the MAP infection and warrants further investigation.

Increasing media copper concentration significantly correlated with reduced PBMC viability. In turn the viability related to the CD14 or 16 expression as well as the NO production. Loss of viability could create a selection bias in the data, as the population of cells that survive are possibly being selected by Cu resistance. As our study used PBMC from five donors this bias may be particularly relevant and suggests larger sample sizes are required. PBMCs that survived Cu treatment may be phenotypically different to those that died, which may impact the resultant surface marker expression levels determined by Flow Cytometry. We attempted to mitigate this effect in the data analysis by including viability into the multivariate mixed linear model. This study did not take into account gut tissue extracellular fluid Cu levels of donor cattle or intracellular monocyte Cu levels. This critical difference could cause the cells to have been incubated at a copper level that was physiologically too high, resulting in loss of viability or a level too low to induce changes in surface marker expression. A study investigating copper toxicity in human PBMCs found the LD50 to be 115 μM copper and saw similar viability percentages to this study (50 to 30% viability) at 115 to 140 μM copper [24]. The cells in our study were treated for 24 h instead of 72 h, perhaps explaining the differences in the viability. Furthermore, Singh et al. 2006 do not specify the Cu anion used; in our study Copper Sulphate (CuSO_4_) was used as it was likely to be less acidic than copper nitrate (Cu(NO_3_)_2_) when in solution. Perhaps the sulphate in the Cu salt affected viability, and not the Cu directly. As the Cu levels are lower than the reported LD50 of Cu in monocytes, it is unlikely the range of copper concentration was too high. Increasing the media Cu concentration from 0 to 16 μM increased the CD14 expression in cows older than four years and equally decreased the CD16 expression levels. There are multiple factors that could have affected this result, including the viability selection bias as previously discussed. Time frame may also have been a variable to consider. PBMC were incubated in the presence of Cu for a total of 48 h, which may have been too long resulting in copper toxicity; equally it could have not long enough to evaluate a change in expression. Other authors polarised human monocytes harvested from the buffy coat of human donors and transitioned into macrophages for 6 days. After cytokine stimulation they incubated for a further 2 days to induce an M1 or M2 phenotype [25]. Another study observing M1 and M2 phenotype plasticity was able to change M1 macrophages into an M2 phenotype after 48 h of stimulation with IFN-γ and LPS [26]. Our study incubated with stimulants for 24 h, therefore the variable of time may have limited the ability to see full M1/M2 changes in the monocytes. Our findings of a higher CD14 and a lower CD16 expression in older cows with higher Cu concentrations suggests a shift in Th1/Th2 alliance of circulating monocytes when exposed to Cu. Physiologically, Cu is dependent on dietary provision, with surplus being stored in the liver. Long term depletion, however, will result in plasma levels below 11 μmol/L, which mimics our current used concentrations. The time dependent aspect deserves further investigation as this study is entirely examining the short-term response. Long term surface marker expression shifts could possibly be more indicative of the correlation between serum Cu and Johne’s disease.

The current findings do raise interesting questions about the mechanism behind the role of Cu on immune cell phenotype plasticity, therefore more in-depth research into this topic is required before entirely ruling the relevance out. In recent years, macrophage immunology research has focused on understanding macrophage phenotype plasticity. In vivo, it is theorised that M1 and M2 phenotypes mark the extremes of a range of phenotype possibilities, and that macrophage phenotypes are plastic in response to changes in the local microenvironment [7]. Early endogenous markers of disease produced by innate immune cells cause a short-term change to monocyte phenotype. For long term change, tissue resident macrophages require signals from antigen specific T cells to generate long lasting specific changes to the phenotype [26]. A study on human macrophages achieved this switch in vitro using LPS and IFN-γ to drive the M1 phenotype, and IL-4 and IL-13 to drive the M2 phenotype [27]. While more research must be conducted to fully understand the mechanisms and range of monocyte phenotype plasticity, particularly in the bovine context, M2 is currently separated into two further groups: an intermediate group and a non-classical (M2) group [13,28].

Johne’s disease has an age-related pathogenesis, with an incubation period of 4 to 14 years. The disease passes through a long subclinical phase before moving onto clinical presentation. The timing of this switch is often around the age range of our samples (4–7 years) [29]. Our findings suggest that age potentially influences the relationship between CD14 expression and Cu concentration. CD14 expression appeared to become positively associated in cows over 4 years, a common age for clinical presentation of Johne’s disease in cattle. In older cattle samples, CD14 expression appeared to increase with Cu concentration, supporting a protective M1 phenotype. In addition, in older cows CD16 expression appeared to decrease as Cu increased, supporting a further move towards M1, a pro-inflammatory phenotype with increased media Cu. However, due to small sample sizes reported here, these findings are indicative rather than absolute and further study is recommended with larger sample sizes stratified by age.

Our findings present the possibility that high serum Cu levels could support a generalised M1 phenotype to enable cattle to remain in the subclinical phase of infection longer. In agricultural animals this would be beneficial, maintaining subclinical throughout their productive lifespan and thereby preventing the spread of MAP infection. The sample size in this study is too small to draw confident conclusions despite statistical significance, and the impact of age and copper on CD14 and CD16 expression must be studied further in a larger population. In addition, further research is required to assess if this relationship between serum Cu levels, macrophage phenotype, Th1 bias and control of MAP exists in vivo. This could be achieved through a randomised clinical trial where Cu supplementation is evaluated in its ability to prevent MAP infected cows progressing to Johne’s disease.

## 5. Conclusions

Our in vitro results suggest that CD14 and CD16 expression of monocytes is modulated under different Cu concentrations in the cell media. In cows over 4 years of age, and with increasing Cu, the CD14 expression appeared to increase and the CD16 expression appeared to decrease suggesting a proxy M1 phenotype. NO production followed CD14 expression, with no additional effect with varying Cu levels. The findings suggests that in older cows, Cu may play an important role to maintain the Th1/Th2 balance favouring towards Th1. This would allow better cellular immunity support in containing intracellular infections, such as MAP in cattle, and as such preventing further progression into JD; conditional on sufficient Cu levels being available in the diet of dairy cows. A randomised clinical trial will be required to determine whether Cu supplementation could prevent MAP infected cows progressing to Johne’s disease.

## Figures and Tables

**Table 1:** Plate design. Cu is copper levels (0, 4, 8 and 16 μM). N is non-stimulated. P is PPD-A stimulus. T is TLR2 ligand stimulus, Pam_3_CSK_4_. U is unstained sample for FACS calibration. Wells denoted X contain no cells or stimulation.

Sample A				Sample B				Sample C				Sample D				Sample E			
Cu	N	P	T	Cu	N	P	T	Cu	N	P	T	Cu	N	P	T	Cu	N	P	T
0	O	O	O	0	O	O	O	0	O	O	O	0	O	O	O	0	O	O	O
4	O	O	O	4	O	O	O	4	O	O	O	4	O	O	O	4	O	O	O
8	O	O	O	8	O	O	O	8	O	O	O	8	O	O	O	8	O	O	O
16	O	O	O	16	O	O	O	16	O	O	O	16	O	O	O	16	O	O	O
U	O	O	X	U	O	O	X	U	O	O	X	U	O	O	X	U	O	O	X

**Table 2:** Associations reported by the backwards stepwise multivariate mixed linear model of CD14/16 expression and Nitric oxide (NO) production of the PBMCs from five dairy cows under varying copper (Cu) levels (0–16 μM). The estimates represent the slope of the linear regression line.

	Outcome Parameter					
Explanatory Parameter	CD14 Expression		CD16 Expression		NO Production	
	Estimate (SD)	*p*-Value	Estimate (SD)	*p*-Value	Estimate (SD)	*p*-Value
Survival (%)	0.13 (0.04)	0.002	−0.38 (0.06)	<0.001	−0.09 (0.02)	<0.001
CD14	#	#	1.34 (0.15)	<0.001	0.27 (0.05)	<0.001
CD16	0.41 (0.05)	<0.001	#	#	ns	Ns
Cu (μM)	−0.31 (0.14)	0.026	ns	Ns	ns	Ns
Age (years)	ns	ns	ns	Ns	ns	Ns
Cu * Age	0.08 (0.03)	0.004	−0.04 (0.02)	0.026	ns	Ns

**#**: not entered in the model, ns: not significant (*p* > 0.05) and no longer in the multivariate mixed linear model. *: interaction term, between copper level and age of the cow.

## Data Availability

The data presented in this study are available on request from the corresponding author.
